# Single nucleotide polymorphism discovery in bovine liver using RNA-seq technology

**DOI:** 10.1371/journal.pone.0172687

**Published:** 2017-02-24

**Authors:** Chandra Shekhar Pareek, Paweł Błaszczyk, Piotr Dziuba, Urszula Czarnik, Leyland Fraser, Przemysław Sobiech, Mariusz Pierzchała, Yaping Feng, Haja N. Kadarmideen, Dibyendu Kumar

**Affiliations:** 1 Division of Functional Genomics in Biological and Biomedical Research, Centre for Modern Interdisciplinary Technologies, Nicolaus Copernicus University, Torun, Poland; 2 Faculty of Biology and Environmental Protection, Nicolaus Copernicus University, Torun, Poland; 3 Faculty of Animal Bioengineering, University of Warmia and Mazury, Olsztyn, Poland; 4 Faculty of Veterinary Medicine, University of Warmia and Mazury, Olsztyn, Poland; 5 Institute of Genetics and Animal Breeding of the Polish Academy of Sciences, Jastrzebiec, Poland; 6 Waksman Institute of Microbiology, Rutgers, The State University of New Jersey, Piscataway Township, NJ, United States of America; 7 Department of Bio and Health Informatics, Technical University of Denmark, Kongens Lyngby, Denmark; University of Florida, UNITED STATES

## Abstract

**Background:**

RNA-seq is a useful next-generation sequencing (NGS) technology that has been widely used to understand mammalian transcriptome architecture and function. In this study, a breed-specific RNA-seq experiment was utilized to detect putative single nucleotide polymorphisms (SNPs) in liver tissue of young bulls of the Polish Red, Polish Holstein-Friesian (HF) and Hereford breeds, and to understand the genomic variation in the three cattle breeds that may reflect differences in production traits.

**Results:**

The RNA-seq experiment on bovine liver produced 107,114,4072 raw paired-end reads, with an average of approximately 60 million paired-end reads per library. Breed-wise, a total of 345.06, 290.04 and 436.03 million paired-end reads were obtained from the Polish Red, Polish HF, and Hereford breeds, respectively. Burrows-Wheeler Aligner (BWA) read alignments showed that 81.35%, 82.81% and 84.21% of the mapped sequencing reads were properly paired to the Polish Red, Polish HF, and Hereford breeds, respectively. This study identified 5,641,401 SNPs and insertion and deletion *(indel)* positions expressed in the bovine liver with an average of 313,411 SNPs and *indel* per young bull. Following the removal of the *indel* mutations, a total of 195,3804, 152,7120 and 205,3184 raw SNPs expressed in bovine liver were identified for the Polish Red, Polish HF, and Hereford breeds, respectively. Breed-wise, three highly reliable breed-specific SNP-databases (SNP-dbs) with 31,562, 24,945 and 28,194 SNP records were constructed for the Polish Red, Polish HF, and Hereford breeds, respectively. Using a combination of stringent parameters of a minimum depth of ≥10 mapping reads that support the polymorphic nucleotide base and 100% SNP ratio, 4,368, 3,780 and 3,800 SNP records were detected in the Polish Red, Polish HF, and Hereford breeds, respectively. The SNP detections using RNA-seq data were successfully validated by kompetitive allele-specific PCR (KASP^TM^) SNP genotyping assay. The comprehensive QTL/CG analysis of 110 QTL/CG with RNA-seq data identified 20 monomorphic SNP hit loci (*CARTPT*, *GAD1*, *GDF5*, *GHRH*, *GHRL*, *GRB10*, *IGFBPL1*, *IGFL1*, *LEP*, *LHX4*, *MC4R*, *MSTN*, *NKAIN1*, *PLAG1*, *POU1F1*, *SDR16C5*, *SH2B2*, *TOX*, *UCP3 and WNT10B*) in all three cattle breeds. However, six SNP loci (*CCSER1*, *GHR*, *KCNIP4*, *MTSS1*, *EGFR* and *NSMCE2*) were identified as highly polymorphic among the cattle breeds.

**Conclusions:**

This study identified breed-specific SNPs with greater SNP ratio and excellent mapping coverage, as well as monomorphic and highly polymorphic putative SNP loci within QTL/CGs of bovine liver tissue. A breed-specific SNP-db constructed for bovine liver yielded nearly six million SNPs. In addition, a KASP^TM^ SNP genotyping assay, as a reliable cost-effective method, successfully validated the breed-specific putative SNPs originating from the RNA-seq experiments.

## Background

With the advancement of high-throughput (HT) NGS technology, transcriptome complexity and its dynamics can now be revealed and explored at different levels. Over the past few years, several sequencing-based technologies have been developed to analyse the transcriptomes in an unprecedented manner, and have revolutionized human and animal genome research [1]. Currently, the most widely used HT-RNA sequencing (RNA-seq) technology utilizes the NGS reads of the entire transcriptome including all transcripts produced in a tissue sample, which was not previously characterized as transcribed sequences and novel isoforms. Moreover, the most important potential applications of RNA-seq technology include the identification of differentially expressed genes (DEGs), co-expressed genes (CEGs) and differences in single nucleotide polymorphism (SNP) variation between experimental groups, such as samples from different (i) tissues (tissue-specific RNA-seq experiments) [2–4], (ii) treatment groups (trait-specific or trait-associated RNA-seq experiments) [5, 6], and (iii) populations (population-based breed-specific RNA-seq experiments) [7–9]. In this study, we utilized an experimental design similar to that described in our recent RNA-seq study on bovine pituitary tissue [9].

Most of the recent studies detecting SNPs using RNA-seq in domestic animals have mainly been focused on the identification of a large number of polymorphisms, with the aim of discovering causative variants involved in phenotypes affecting economic traits of interest in different domestic animal species, *e*.*g*., sheep [10], goat [11], pig [12], horse [13], chicken [14] and cattle [15–20]. Furthermore, SNP markers have increasingly been used in cattle breeding improvement programmes, *e*.*g*., marker-assisted selection (MAS), gene-assisted selection (GAS) and genomic selection (GS) [21–23], as a means of conventionally improving phenotypic selection. It is noteworthy that SNP detection for economic traits has great potential in the genetic improvement of cattle through the implementation of MAS, GAS and GS programmes, which have been highly recommended to the global cattle breeding programme.

In this study, we have chosen to perform RNA-seq on bovine liver tissue because of its highly robust metabolic activity [24], and because it is one of the most common target organ sites for body growth, feed utilization or feed efficiency and developmental trait assessments [25]. It should be emphasized that the liver has a major influence on the genetic improvement of production trait variation [26]. Even though SNPs have been identified in several RNA-seq experiments in cattle [8, 9, 15–20, 27], to our knowledge, there is a lack of studies that detect putative SNPs in bovine liver tissue. In this study, we used the NGS-based RNA-seq technology to characterize and compare bovine liver transcriptomes of the Polish Red, Polish HF and Hereford breeds, including the detection and construction of breed-specific SNP-databases (SNP-dbs), and analysis of QTL/CG and single nucleotide variation.

## Results

### mRNA sequencing and read alignment

mRNA sequencing of bovine liver at single-nucleotide resolution was carried out using two biological replicates of poly(A)-enriched mRNA of young bulls aged 6, 9, and 12 months from three cattle breeds. These mRNA samples were first converted into barcoded strand-specific dUTP RNA-seq libraries, followed by HT sequencing on the Illumina NextSeq 500 sequencer. The HT sequencing produced a total of 107,114,4072 raw paired-end reads with a length of 156 bases. The reads were de-multiplexed to assign reads to each sequenced sample according to its index. The FASTQ sequence dataset of each library (Table 1) was submitted to the NCBI-SRA database with NCBI-SRA experiment number SRS1296732 (https://www.ncbi.nlm.nih.gov/sra?linkname=bioproject_sra_all&from_uid=312148).

**Table 1:** Description of submitted FASTQ sequences of bovine liver of all 18 young bulls from three cattle breeds using RNA-seq.

Breed	Age	Animal ID	Library name	SRA Run	MBases	Mbytes	SRA Experiment	SRA accession no.	FastQ file size
Polish RED	6m	6938	CP19	SRR3176171	8488	4041	SRX1590547	SRX1590547	1 ILLUMINA (NextSeq 500) run: 28.8M spots, 8.9G bases, 3.9Gb downloads
	6m	6944	CP20	SRR3180685	8015	3817	SRX1595483	SRX1595483	1 ILLUMINA (NextSeq 500) run: 27.4M spots, 8.4G bases, 3.7Gb downloads
	9m	6919	CP21	SRR3180686	10924	4988	SRX1595484	SRX1595484	1 ILLUMINA (NextSeq 500) run: 39.4M spots, 11.5G bases, 4.9Gb downloads
	9m	6924	CP22	SRR3176235	16686	7922	SRX1590605	SRX1590605	1 ILLUMINA (NextSeq 500) run: 56.4M spots, 17.5G bases, 7.7Gb downloads
	12m	9951	CP23	SRR3176228	4848	2304	SRX1590598	SRX1590598	1 ILLUMINA (NextSeq 500) run: 16.5M spots, 5.1G bases, 2.3Gb downloads
	12m	9965	CP24	SRR3176249	6003	2845	SRX1590619	SRX1590619	1 ILLUMINA (NextSeq 500) run: 20.7M spots, 6.3G bases, 2.8Gb downloads
Polish-HF	6m	9933	CP27	SRR3176230	3902	1834	SRX1590600	SRX1590600	1 ILLUMINA (NextSeq 500) run: 13.4M spots, 4.1G bases, 1.8Gb downloads
	6m	9938	CP28	SRR3176240	5686	2661	SRX1590610	SRX1590610	1 ILLUMINA (NextSeq 500) run: 20.2M spots, 6G bases, 2.6Gb downloads
	9m	8603	CP29	SRR3176241	8058	3710	SRX1590611	SRX1590611	1 ILLUMINA (NextSeq 500) run: 28.6M spots, 8.4G bases, 3.6Gb downloads
	9m	8602	CP30	SRR3176233	7018	3225	SRX1590603	SRX1590603	1 ILLUMINA (NextSeq 500) run: 25M spots, 7.4G bases, 3.1Gb downloads
	12m	7140	CP31	SRR3176229	5284	2423	SRX1590599	SRX1590599	1 ILLUMINA (NextSeq 500) run: 18.8M spots, 5.5G bases, 2.4Gb downloads
	12m	7037	CP32	SRR3176232	5332	2538	SRX1590602	SRX1590602	1 ILLUMINA (NextSeq 500) run: 18.9M spots, 5.6G bases, 2.5Gb downloads
Herford	6m	4051	CP35	SRR3176234	6320	2918	SRX1590604	SRX1590604	1 ILLUMINA (NextSeq 500) run: 22.4M spots, 6.6G bases, 2.9Gb downloads
	6m	4049	CP36	SRR3180682	9948	4600	SRX1595481	SRX1595481	1 ILLUMINA (NextSeq 500) run: 35.2M spots, 10.4G bases, 4.5Gb downloads
	9m	4069	CP37	SRR3176238	10924	4988	SRX1590608	SRX1590608	1 ILLUMINA (NextSeq 500) run: 39.4M spots, 11.5G bases, 4.9Gb downloads
	9m	4072	CP38	SRR3176236	4561	2115	SRX1590606	SRX1590606	1 ILLUMINA (NextSeq 500) run: 15.7M spots, 4.8G bases, 2.1Gb downloads
	12m	4005	CP39	SRR3176237	6620	3029	SRX1590607	SRX1590607	2 ILLUMINA (NextSeq 500) run: 35.7M spots, 10.8G bases, 4.1Gb downloads
	12m	3988	CP40	SRR3176250	15241	7047	SRX1590620	SRX1590620	1 ILLUMINA (NextSeq 500) run: 54.8M spots, 16G bases, 6.9Gb downloads

Using the Burrows-Wheeler Aligner (BWA) program under default conditions, the breed-specific liver transcripts were mapped to the bovine reference genome (UMD3.1 assembly plus Y chromosome). Our read alignment results showed that 98.55% sequencing reads (105,567,9630) were successfully aligned to the UMD3.1 bovine reference genome. Furthermore, the acquired BWA mapping profile results showed that 81.35%, 82.81% and 84.21% of the mapped sequencing reads were properly paired for the Polish Red, Polish HF, and Hereford breeds, respectively (Tables 2–4).

**Table 2:** Transcriptome mapping profile of liver tissue from the Polish Red cattle breed aligned to bovine reference UMD3.1 genome assembly.

Age	Total	Mapped	Paired in sequencing	read1	read2	Properly paired	With itself and mate mapped	Singletons	With mate mapped to a different chr	With mate mapped to a different chr (map Q> = 5)
6m	129083165	125618965	129083165	64058541	65024624	104968754	123975096	1643869	5079498	2731309
9m	129828806	127331756	129828806	64383140	65445666	102598750	126102410	1229346	6200605	3836617
12m	86157331	84520505	86157331	42810031	43347300	66969668	83577919	942586	3987897	2423912
Total	345069302	337471226	345069302	171251712	173817590	274537172	333655425	3815801	15268000	8991838

**Table 3:** Transcriptome mapping profile of liver tissue from the Polish HF cattle breed aligned to bovine reference UMD3.1 genome assembly.

Age	Total	Mapped	Paired in sequencing	read1	read2	Properly paired	With itself and mate mapped	Singletons	With mate mapped to a different chr	With mate mapped to a different chr (map Q> = 5)
6m	78638726	77583615	78638726	38885550	39753176	62926014	77133629	449986	2768888	727097
9m	125158864	123855247	125158864	61933615	63225249	102019446	123246170	609077	3420578	922272
12m	86243971	85516298	86243971	42873038	43370933	72706552	85171918	344380	2146832	568684
Total	290041561	286955160	290041561	143692203	146349358	237652012	285551717	1403443	8336298	2218053

**Table 4:** Transcriptome mapping profile of liver tissue from the Hereford cattle breed aligned to bovine reference UMD3.1 genome assembly.

Age	Total	Mapped	Paired in sequencing	read1	read2	Properly paired	With itself and mate mapped	Singletons	With mate mapped to a different chr	With mate mapped to a different chr (map Q> = 5)
6m	131040830	129659186	131040830	64929303	66111527	110158835	129106337	552849	3702495	777228
9m	126894781	125680372	126894781	62842854	64051927	104811545	125092492	587880	3869208	938567
12m	178097598	175913686	178097598	88097058	90000540	148218515	175078651	835035	5065145	1323493
Total	436033209	431253244	436033209	215869215	220163994	363188895	429277480	1975764	12636848	3039288

### SNP discoveries in cattle breeds

#### Breed-specific raw SNP-db records

Using the SAMtool package, a total of 5,641,401 (~5.6 million) breed-specific SNPs and *indel* positions expressed in the bovine liver were detected with the RNA-seq reads, with an average of 313,411 (~ 0.31 million) SNPs and *indels* per young bull (Table 5). Breed-wise, this raw SNP-db comprised 1,995,571 (35.4%), 1,556,048 (27.6%), and 2,089,782 (37%) SNPs and *indels* for the Polish Red, Polish HF, and Hereford breeds, respectively. Following the removal of the *indel* mutations, a total of 1,953,804 (35.3%), 1,527,120 (27.6%), and 2,053,184 (37.1%) raw SNPs expressed in bovine liver were recovered from the Polish Red, Polish HF, and Hereford breeds, respectively. Using the SAMtool package, single-base substitutions (SNPs) and small *indels* were also identified. In this study, a total of 41,767, 36,604 and 28,934 *indel* mutations were identified in the Polish Red, Polish HF, and Hereford breeds, respectively.

**Table 5:** Construction of breed-specific raw SNP-db of bovine liver transcriptome.

Breed	Age	Raw SNPs-db			Source files
		Only *indels*	Only SNPs	SNPs and *indel*s	
Hereford	6 months	6396	330413	336808	S1 Table
Hereford	6 months	6367	344246	350612	S2 Table
Hereford	9 months	3594	290198	293791	S3 Table
Hereford	9 months	4828	297236	302063	S4 Table
Hereford	12 months	6781	361527	368307	S5 Table
Hereford	12 months	8638	429564	438201	S6 Table
Sub-Total		36604	2053184	2089782	
Polish Red	6 months	6090	346054	352144	S7 Table
Polish Red	6 months	4804	289124	293928	S8 Table
Polish Red	9 months	12049	325913	337962	S9 Table
Polish Red	9 months	6857	395364	402221	S10 Table
Polish Red	12 months	5778	256349	262127	S11 Table
Polish Red	12 months	6189	341000	347189	S12 Table
Sub-Total		41767	1953804	1995571	
Polish HF	6 months	2922	184589	187510	S13 Table
Polish HF	6 months	3496	195466	198961	S14 Table
Polish HF	9 months	6142	297467	303608	S15 Table
Polish HF	9 months	5982	322852	328833	S16 Table
Polish HF	12 months	5133	277258	282390	S17 Table
Polish HF	12 months	5259	249488	254746	S18 Table
Sub-Total		28934	1527120	1556048	
Sum of all Totals		107305	5534108	5641401	

#### SNP distribution in Venn plot

**Fig 1:**
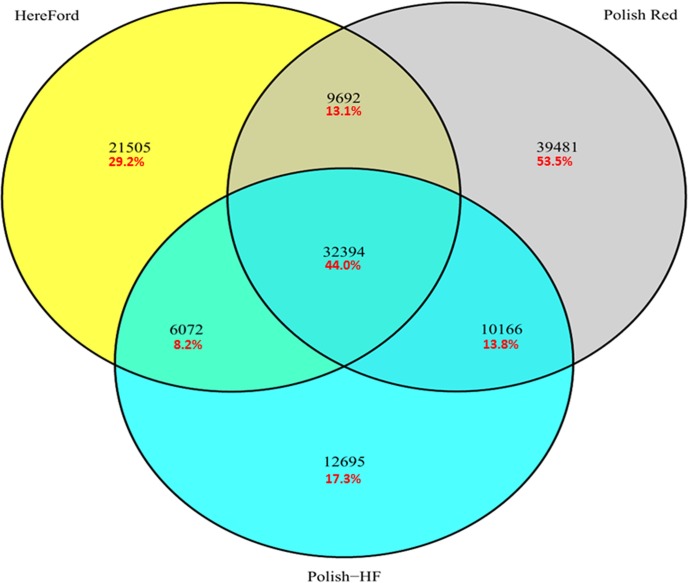
Venn diagram showing the number of SNPs segregating in Polish Red, Polish HF and Hereford cattle breeds

In our initial SNPs analysis, a stringent filtering parameter of read count with a minimum depth of ≥5 SNP reads that support the polymorphic nucleotide base and existed in both replicates was utilized to allow the identification of approximately 0.8 million SNPs among the three cattle breeds as shown in Venn diagram (Fig 1).

#### Breed-specific SNP-db records

For the detection of breed-specific putative SNPs expressed in bovine liver, only the records in the raw SNP-db (S1–S18 Tables) were combined into one file to construct a highly reliable SNP-db with 84,701 SNP hit records. Three highly reliable breed-specific SNP-dbs comprising 31,562 (37.27%), 24,945 (29.45%) and 28,194 (33.28%) SNP records were constructed for the Polish Red, Polish HF, and Hereford breeds, respectively (S19–S21 Tables).

**Error removals:** During SNP detection using SAMtool, some records were observed as more than one SNP mutation. Such records were considered as error records and were excluded from the SNP-db. In our study, a total of 4381, 1164 and 1202 such error records in the Polish Red, Polish HF and Hereford breeds were observed and excluded. After removal of the error records, a total of 27,182, 23,781 and 26,992 SNP-db records were recovered from the Polish Red, Polish HF and Hereford breeds, respectively (S22–S24 Tables).

#### SNP filtering

We utilized the stringent parameter of a minimum depth of 10 SNP reads that support the polymorphic nucleotide base with a SNP ratio of 100%, because the SNP filtering criteria of ≥10 SNP reads with a SNP ratio of 100% could cover and explain all the HT SNP variations compared with the SNP filtering criteria of ≥10 SNP reads with a SNP ratio of 90% [9].

Initially, for the SNP filtering analysis, we utilized stringent parameters with a minimum depth of ≥10 SNP reads that support the polymorphic nucleotide base and identified 15,197, 11,346 and 12,455 SNP records for the Polish Red, Polish HF, and Hereford breeds, respectively (S25–S27 Tables). Similarly, by utilizing stringent parameters of 100% SNP ratios, we identified 10,206, 9,684 and 9,778 SNP records for the Polish Red, Polish HF, and Hereford breeds, respectively (S28–S30 Tables). Finally, the combination of both stringent filtering parameters of ≥10 reads, and a 100% SNP ratio, yielded a total of 4,368, 3,780 and 3,800 SNPs records for the Polish Red, Polish HF, and Hereford breeds, respectively (S31–S33 Tables).

#### Breed comparison

Comparison of the breed-specific SNP records resulted in the identification of common and unique SNPs. For each breed, three comparisons were made to find common and unique SNPs within the investigated breeds. For example, i) a comparison between breed-1 (Polish Red) with breed-2 (Polish HF); ii) a comparison between breed-1 and breed-3 (Hereford); and iii) a comparison of breed-1 to both breed-2 and breed-3.

In this study, a total of 50 and 81 unique SNP loci were identified in the Polish Red breed that were not present in either the Polish HF or Hereford breeds, respectively (S34 and S35 Tables). In addition, only six unique SNP records were identified in the Polish Red breed that was not detected in either Polish HF or Hereford breeds (S36 Table).

Similarly, the SNP data comparison of the Polish HF breed to other breeds resulted in the identification of 7 and 22 unique SNPs that were not present in the Polish Red or Hereford breeds, respectively (S37 and S38 Tables, respectively). However, no single unique SNP record was identified in the Polish HF breed that was not present in either the Polish Red or Hereford breeds, respectively (S39 Table). Finally, the SNP data comparison of the Hereford breed to other breeds identified a total of 80 and 41 unique SNPs that were not detected in Polish Red or Polish HF breeds, respectively (S40 and S41 Tables). Furthermore, a total of nine unique SNP records of the Hereford breed were identified, which were not present in either the Polish Red or Polish HF breeds (S42 Table).

#### *De novo* SNPs

Deep and extensive SNP analysis provided evidence that certain regions of the *Bos taurus* genome were still unknown (base = N, according to recent mapping to UMD3.1). Using the SNP filtering criteria (≥10 SNP reads with SNP ratio of 100%), a total of 217, 193 and 265 best candidates of *de novo* SNP reads were identified for the Polish Red, Polish HF, and Hereford breeds, respectively (S43–S45 Tables).

### Breed-specific SNP discovery and QTL/CG analysis

For the QTL/CG analysis on identified breed-specific SNP data, a set of 110 QTL/CG loci (http://www.animalgenome.org/cgi-bin/QTLdb/index) (S46 Table) was comprehensively investigated for each breed and ages of the young bulls (S47–S52 Tables). We summarized, for each breed, the chromosomal locations and SNP locations of identified putative SNP loci hits of RNA-seq data on 110 potential CGs from the bovine QTL-db (S47–S49 Tables). In S50–S52 Tables, we further summarized the numbers of putative SNP hits of RNA-seq data, identified within breed (ages) and between breeds. Overall, we have identified i) monomorphic breed-specific SNPs, and ii) highly polymorphic breed-specific SNPs within the investigated 110 QTL/CGs loci (S50–S52 Tables).

#### Identification of breed-specific monomorphic SNP loci in bovine QTL/CG db

Breed-wise, a total of 32, 28, and 29 monomorphic SNP hits on 110 QTL/CG genes were identified in the Polish Red, Polish HF and Hereford breeds, respectively. Among the identified monomorphic SNP hit loci, 20 CGs (*CARTPT*, *GAD1*, *GDF5*, *GHRH*, *GHRL*, *GRB10*, *IGFBPL1*, *IGFL1*, *LEP*, *LHX4*, *MC4R*, *MSTN*, *NKAIN1*, *PLAG1*, *POU1F1*, *SDR16C5*, *SH2B2*, *TOX*, *UCP3 and WNT10B*) were monomorphic to all investigated breeds. Furthermore, SNP hits in the *BMP8B*, *GHSR*, and *RFX6* CGs were monomorphic only to the Polish Red and Polish HF breeds, whereas SNP hits in the *DLK2*, *MYF5*, and *PROP1* CGs were monomorphic to both the Polish HF and Hereford breeds. Monomorphic SNP loci in the *BTG4*, *NPM1*, *NPy* and *SIX3* CGs were detected in the Polish Red and Hereford breeds. In addition, SNP hits in the *AMPD1*, *CAPN3*, *MYF6* and *SDR16C6* CGs were monomorphic only to the Polish Red breed, while a SNP hit in the *GDP10* CG was monomorphic only to the Polish HF breed, and SNP hits in the *GHRHR* and *IGFN1* CGs were monomorphic only to the Hereford breed.

#### Identification of highly polymorphic breed-specific SNP loci in the bovine QTL/CG db

Highly polymorphic breed-specific SNP loci were summarized as top-10 and top-20 SNP hits (S50–S52 Tables). Among the top 10 SNP hits, five CGs (*CCSER1*, *GHR*, *KCNIP4*, *MTSS1*, *EGFR* and *NSMCE2*) were highly polymorphic to all three cattle breeds. Furthermore, SNP hits in the *IGF-I* CG were highly polymorphic to the Polish Red and Polish HF breeds, SNP hits in the *CAST* CG were highly polymorphic to the Polish HF and Hereford breeds, and SNP hits in the *LEPR* CG were highly polymorphic to the Polish Red and Hereford breeds. In addition, SNP hits in the *IGF2R* and *MAP3K5* CG loci were highly polymorphic to the Polish Red breed, SNP hits in the *INSIG1* and *ATP6V1B2* CG loci were highly polymorphic to the Polish HF breed, and SNP hits in the *PRLR*, *IGFBP2* CG loci were highly polymorphic to the Hereford breed.

### Phylogenetic analysis of the breed-specific SNP-db

**Fig 2:**
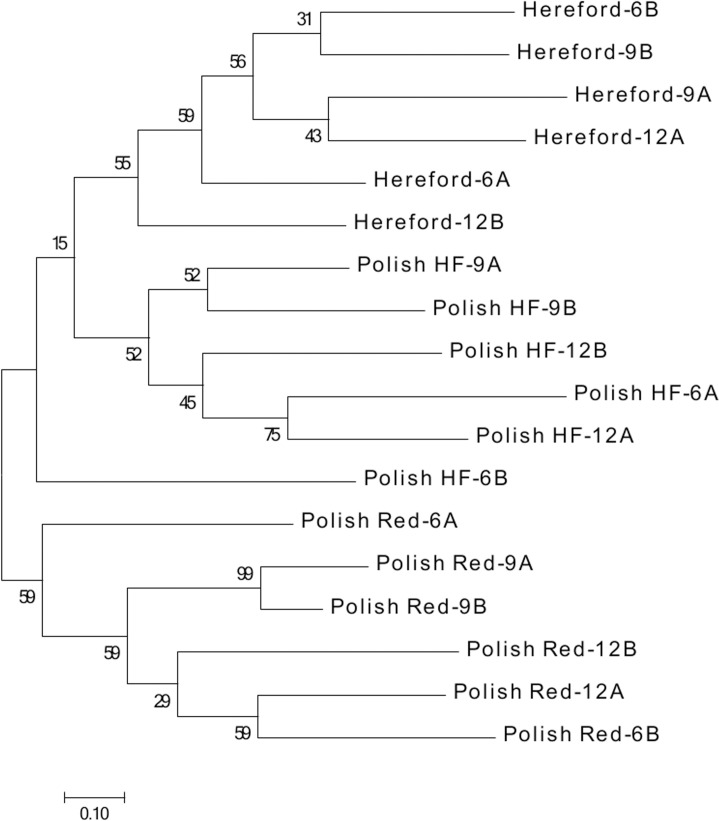
The phylogenetic relationship among RNA-seq samples of bovine liver using Maximum likelihood method based on the JTT matrix-based model. An unrooted phylogeny tree of 18 bulls’ samples representing developmental ages 6 months (6A, 6B), 9 months (9A, 9B), and 12 months (12A, 12B) of Polish Red, Polish-HF and Hereford breeds. The percentage of trees in which the associated taxa clustered together is shown next to the branches. All nodes were robust at 100% bootstrap support. The scale bar denotes substitutions per site.

Additionally, a phylogenetic tree was constructed using a JTT matrix-based model with superior log maximum likelihood values [28] and molecular evolutionary genetics analysis version 7 (MEGA7) software [29] to examine the close relationship of the investigated bulls within and between breeds. Results showed that all three cattle breeds were separated from each other, but clustered together for each breed. The percentage of trees in which the associated taxa clustered together is shown next to the branches (Fig 2).

### Breed-specific SNP validation

#### Selection of nine breed-specific putative SNPs

A breed-specific SNP validation experiment was carried out using a subset of nine putative SNPs derived from the Polish Red (n = 2), Polish HF (n = 4) and Hereford (n = 3) breeds, on single-plex KASP^TM^ genotyping assays (LGC Genomics) based on fluorescently labelled allele-specific PCR primers (Table 6). The selection of nine putative breed-specific SNPs was performed according to the RNA-seq experimental results (S53 Table). We selected three SNPs that were uniquely expressed in only one cattle breed (*CTNS* and *P4HA2* SNP gene loci specific to the Polish Red breed and *IQGAP2* SNP gene locus specific to the Hereford breed), and the remaining six SNP gene loci that were specific to all the breeds (*GHR*, *IGF2R*, *IGF2BP3*, *IGFBP4* SNP gene loci specific to the Polish HF breed, and *GHR and IGF2* SNP gene loci specific to the Hereford breed, respectively). Based on the RNA-seq experimental results, detailed information of the primer design of the nine selected breed-specific SNP loci, such as the candidate genes and their symbols, genome locations, SNP positions, UMD 3.1 chromosome and gene map positions, and both 5`and 3`flanking regions of 200 bp sequences at the SNP mutation site, is illustrated in S54 Table.

**Table 6:** The distribution (n) of investigating young bulls in a breed-specific experimental design representing bovine liver transcriptome.

Breeds	6 months	9 months	12 months	Total
RNA-seq experiment				
Hereford	2	2	2	6
Polish HF	2	2	2	6
Polish Red	2	2	2	6
Total	6	6	6	18
SNP validation experiment				
Hereford	5	5	5	15
Polish HF	5	4	5	14
Polish Red	5	5	5	15
Total	15	14	15	44

The selected subset of the nine breed-specific putative SNP markers from the Polish Red, Polish HF and Hereford breeds worked well in KASP^TM^ SNP genotyping assay and did not reveal either non-amplification or ambiguous clustering, except for a few samples due to poor DNA quality (Figs 3–11).

**Fig 3:**
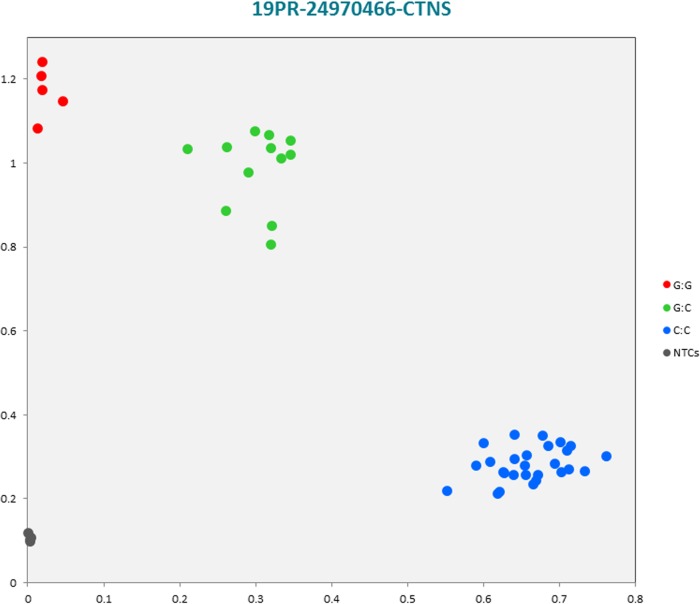
KASP^TM^ SNP genotyping assay of BTA19_24970466 locus of Polish Red *CTNS* gene showing the data for single KASP^TM^ assays on a single cluster plot.

**Fig 4:**
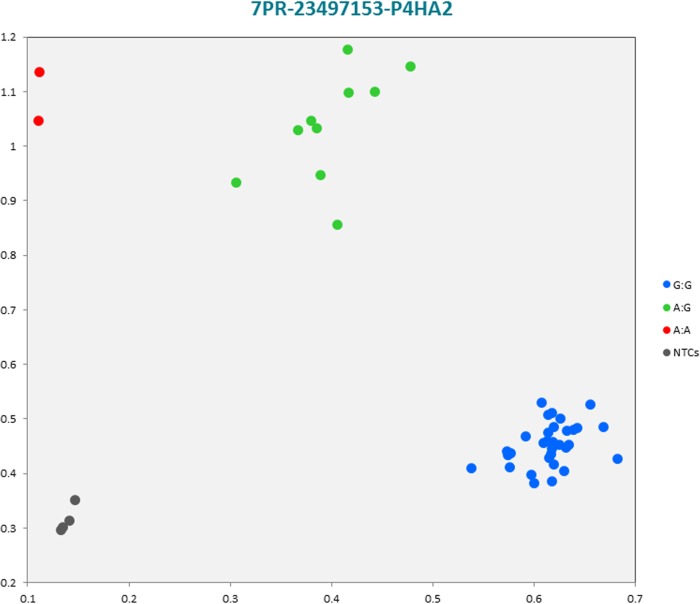
KASP SNP^TM^ genotyping assay of BTA7_23497153 locus of Polish Red *P4HA2* gene showing the data for single KASP^TM^ assays on a single cluster plot.

**Fig 5:**
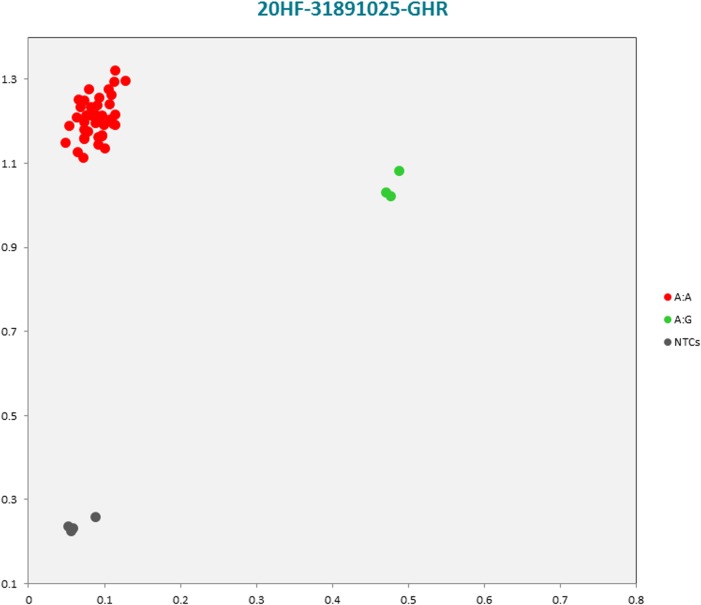
KASP^TM^ SNP genotyping assay of BTA20_31891025 locus of Polish HF *GHR* gene showing the data for single KASP^TM^ assays on a single cluster plot.

**Fig 6:**
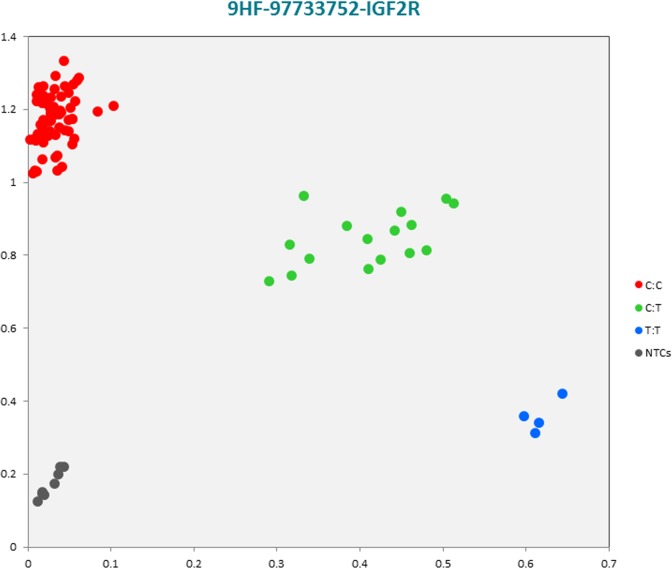
KASP^TM^ SNP genotyping assay of BTA9_97733752 locus of Polish HF *IGF2R* gene showing the data for single KASP^TM^ assays on a single cluster plot.

**Fig 7:**
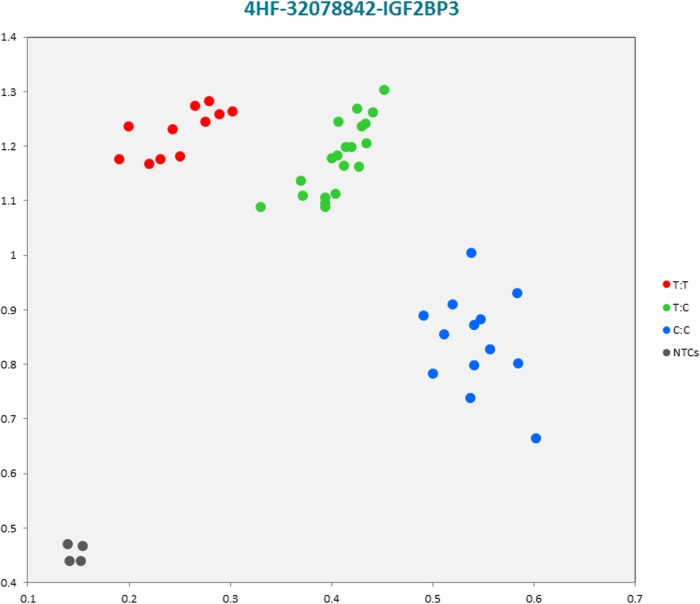
KASP^TM^ SNP genotyping assay of BTA4_32078842 locus of Polish HF *IGF2BP3* gene showing the data for single KASP^TM^ assays on a single cluster plot.

**Fig 8:**
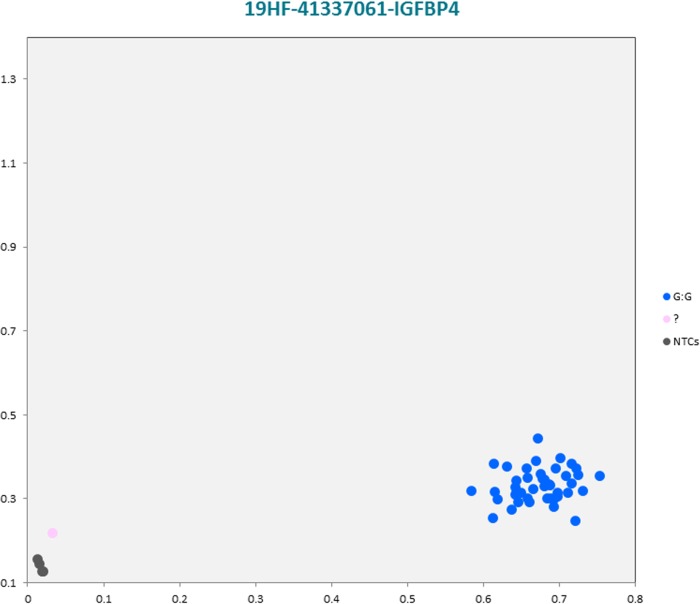
KASP^TM^ SNP genotyping assay of BTA19_41337061 locus of Polish HF *IGFBP4* gene showing the data for single KASP^TM^ assays on a single cluster plot.

**Fig 9:**
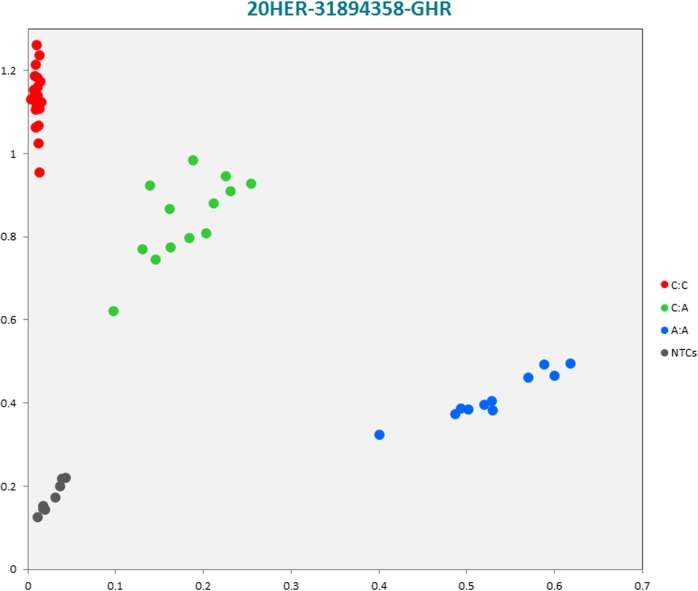
KASP^TM^ SNP genotyping assay of BTA20_31894358 locus of Hereford *GHR* gene showing the data for single KASP^TM^ assays on a single cluster plot.

**Fig 10:**
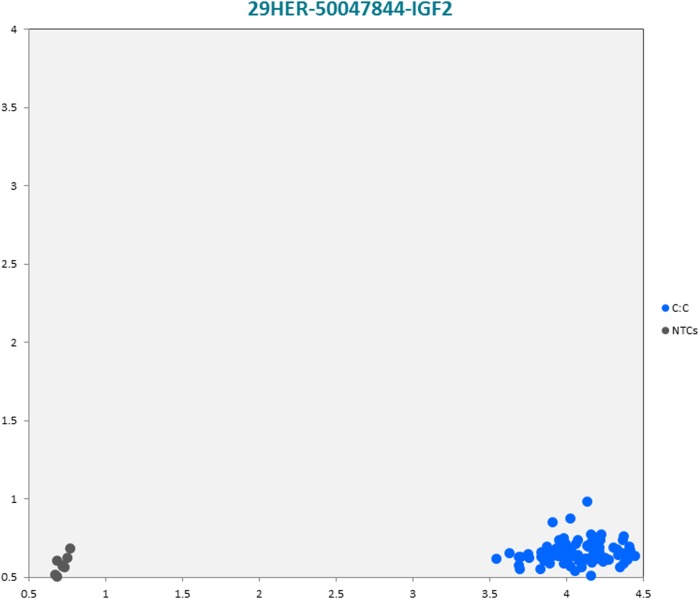
KASP^TM^ SNP genotyping assay of BTA29_50047844 locus of Hereford *IGF2* gene showing the data for single KASP^TM^ assays on a single cluster plot.

**Fig 11:**
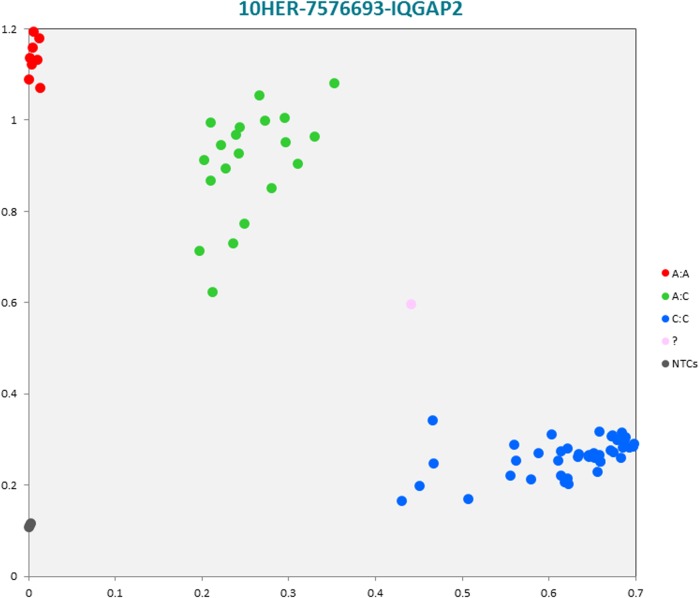
KASP^TM^ SNP genotyping assay of BTA10_7576693 locus of Hereford *IQGAP2* gene showing the data for single KASP^TM^ assays on a single cluster plot.

#### KASP^TM^ SNP assay analysis

Initially, an additional analysis for the estimation of a direct relationship between breed and the detected SNP polymorphisms was performed using the PROC MIXED SAS 9.2 package with age as a random effect. Two SNP markers, *IGFBP4* (Polish HF) and *IGF2* (Hereford) genes were identified as homozygous at the SNP loci, and therefore were excluded for further statistical SNP validation analysis (S55 Table). The results of the remaining seven SNP loci did not show any significant association between age and SNPs selected for the validation. However, there were significant and highly significant associations between breed and the selected SNPs (S56 Table). Furthermore, a chi-square analysis showed significant differences in genotypes and allele frequencies for the *CTNS* gene SNP locus specific to the Polish Red breed, *IGF2R* gene SNP locus specific to the Polish HF breed, and the *GHR* and *IQGAP2* genes SNP loci specific to the Hereford breed (S57 Table).

#### Statistical analysis using Genepop software

Using the Fisher's Exact Probability test, the genetic differentiation of SNP alleles and SNP genotype results revealed significant differences in SNP allele frequencies for the *CTNS*, *IGF2R*, *GHR (*Polish HF breed), *IGF2BP3*, *GHR (*Hereford breed) and *IQGAP2* SNP loci (S58 Table). Regarding the genotype frequencies, significant differences were observed in the *CTNS*, *P4HA2*, *IGF2R*, *GHR (*Polish HF breed), *IGF2BP3*, *GHR (*Hereford breed), and *IQGAP2* SNP loci (S59 Table) in all investigated cattle breeds. Moreover, the genetic differentiation comparison of the SNP alleles (S60 Table) and genotypes (S61 Table) in all SNP loci and all investigated cattle breeds were also performed, using Fisher's Exact Probability test and Fisher's Exact G test. Results showed highly significant differences in the SNP alleles (S60 Table) and SNP genotypes (S61 Table) among breeds to validate the breed-specific SNP markers. For SNP validation statistics using a Markov chain method, the selected SNP markers were further examined by testing the deviation from Hardy Weinberg equilibrium (HWE) for each SNP locus (S62 Table), and for each breed population (S63 Table). The results based on genetic differentiation of the investigated SNPs across all loci showed significant differences in the *CTNS*, *IGF2R*, *IGF2BP3*, *GHR* (Polish HF), and *IQGAP2* SNP loci (S62 Table). Furthermore, the results based on genetic differentiation of the investigated SNPs across all breeds revealed significant differences among them (S63 Table).

## Discussion

### mRNA sequencing and read alignment

RNA-seq technology has great potential in identifying genetic variation at many loci, with respect to SNP polymorphisms and gene expression patterns across different organ tissues. In the present study, we identified approximately 107 million raw paired-end reads with an average length of 156 bases in bovine liver tissue compared to 113 million raw paired-end reads detected in the bovine pituitary gland [9]. This variation in the RNA-seq yield from different organ tissues might be due to technical variation, such as differences in the quality and quantity of the RNA recovered during tissue-specific sample preparations, batch effects in library preparation [30, 31], flow cell and lane effects caused by the Illumina sequencing platform, or adapter bias [32, 33]. However, both the liver and pituitary gland tissues revealed higher percentages (99.4% and 98.5%, respectively) of read alignment to the UMD3.1 bovine reference genome. Furthermore, using the BWA program under default conditions, showed that 94.39%, 93.04% and 83.46% of the mapped sequencing reads of the pituitary gland tissues were properly paired for the Polish Red, Polish HF, and Hereford breeds, respectively [9].

The FASTQ sequences of both tissues were submitted with the NCBI-SRA experiment number SRS1296732 (http://www.ncbi.nlm.nih.gov/sra?linkname=bioproject_sra_all&from_uid=312148). Currently, there are 24 SRA records (FASTQ sequence dataset) for bovine liver tissue at the SRA NCBI database [34] (https://trace.ncbi.nlm.nih.gov/Traces/sra/sra.cgi?view=studies&term=(bos%20taurus%20liver)%20NOT%20cluster_dbgap%5BPROP%5D)).

### SNP discovery in cattle breeds

One of the potential applications of the RNA-seq technology is to identify several thousands of SNPs and as well as to construct the tissue-specific SNP datasets. In our study, we constructed breed-specific SNP-db records for bovine liver tissue, with more than 5.6 million SNP records compared to 13.7 million SNP-db records for the bovine pituitary gland tissue [9]. The differences in the SNP data yield in tissue-specific RNA-seq experiments in the bovine liver and pituitary gland might be due to pre- and post-quality assessment of read alignments, *i*.*e*., quality control (QC) metrics before alignment [35]. In most SNP discoveries based on bovine RNA-seq studies [7–9, 16–20], selection of stringent parameters in the SNP filtering process is undoubtedly a critical task for identifying the most reliable novel putative SNPs. Using the stringent parameter of a minimum depth of 10 SNP reads that support the polymorphic nucleotide base with a SNP ratio of 100%, we identified 20,573, 31,978 and 30,052 breed-specific SNP records for bovine pituitary gland in the Polish Red, Polish HF and Hereford breeds, respectively [9]. However, in the current study, we identified 31,562, 24,945 and 28,194 breed-specific SNP records for bovine liver tissue in the Polish Red, Polish HF and Hereford cattle breeds, respectively.

It should be emphasized that, in cattle breeding practices, identification of breed-specific gene-associated SNPs can serve as suitable markers for trait-associated studies and can be effectively utilized in genomic selection (GS) programmes [36]. In our study, we investigated 110 QTL/CG loci to identify novel putative gene-associated SNPs within a breed, with respect to the animal’s age, and between breeds. Results based on within-breed QTL/CG analysis revealed that approximately one third of the selected QTL/CG loci were monomorphic in all investigated breeds. These findings indicate that the dairy and beef cattle breeds might have certain genetic selection signatures due to fixation of certain genotypes (monomorphic SNPs). It is noteworthy that, between breeds, the QTL/CG analysis identified *CCSER1*, *GHR*, *KCNIP4*, *MTSS1*, *EGFR* and *NSMCE2* gene loci as highly polymorphic. Moreover, analysis of the 76 QTL/CGs in bovine pituitary gland RNA-seq data demonstrated that *KCNIP4*, *CCSER1*, *DPP6*, *MAP3K5 and GHR* genes loci were highly polymorphic in all the investigated cattle breeds [9]. These results indicate that there is a still an abundant genetic variation in the Polish dairy and beef cattle breeds due to the high degrees of polymorphism in the CGs of economically important traits, which could be exploited in GS and other breeding programmes.

In cattle, many studies have utilized QTL/CG analysis with respect to whole genome sequence (WGS) data, genome-wide association study (GWAS) data and expression quantitative trait loci (eQTL) data, to investigate the causal relationships of variant-trait and variant-gene expression, respectively [37–40]. Using human and bovine data, Seo *et al*. [5] have successfully demonstrated the application of the association test approach based on RNA-seq analysis to identify trait associated genes (TAGs).

In this study, we have validated and revealed that breeds of the investigated bulls were separated from each other using an ITT matrix based maximum likelihood model [28] and MEGA 7 [29]. With regards to bovine pituitary gland tissue, we successfully utilized another phylogenetic analysis method using the SNPphylo model [41], which revealed that the three cattle breeds were clustered together for each breed and were separated from each other [9].

Finally, the utilization of KASP^TM^ genotyping assay technology in our SNP validation experiment has confirmed the presence of seven breed-specific SNP markers, which are expressed in bovine liver tissue. In addition, a similar SNP experiment has been successfully performed to validate the expression of the seven SNP markers in bovine pituitary tissue [9]. Thus, the SNP experiment using KASP^TM^ genotyping assay technology is an effective and reliable method to validate RNA-seq in various bovine organ tissues.

## Conclusions

Breed-specific SNP discovery using NGS-based RNA-seq in bovine liver tissue has been reported to provide a global view of the complexity of the bovine liver transcriptome. Our study has developed three breed-specific SNP-dbs based on expressed genes in the bovine liver, which might provide valuable resources for trait-associated genomic and genome-wide association studies. Our study has demonstrated the utility of QTL/CG analysis on RNA-seq SNP data to identify putative trait-associated SNPs from the bovine QTL-db. Our phylogenetic analyses have shown that all three cattle breeds were separated from each other long ago and that each breed is represented as unique genetic clusters. The transcriptome sequencing (RNA-seq) technique employed in the current study is similar to the Genotyping-by-Sequencing (GBS) and it has good potential for improving the accuracy of genomic selection because it involves several millions of SNPs covering the entire transcriptome, which increases the chance of identifying QTLs or quantitative trait nucleotides (QTNs) in linkage disequilibrium with SNPs. Furthermore, these results suggest that there are wide differences in the liver transcriptomes between the breeds, which could be useful to study the mechanisms underlying genetic variability in meat quality and other production traits in cattle.

## Materials and methods

### Experimental design

The bovine liver tissue samples were collected from 18 young bulls of three cattle breeds (Table 6) stationed at the Institute of Genetics and Animal Breeding (IGAB), Polish Academy of Science (PAS), Jastrzębiec, Poland. The investigated animals were selected randomly, and after the purchase at birth, they were reared in the experimental farm of IGAB, PAS, Jastrzębiec, Poland, with uniform environmental and feeding conditions. After slaughtering, the collected liver tissues were immediately kept in liquid nitrogen, and stored at -80°C. All procedures involving animals were conducted in accordance with the guiding principles for the care and use of research animals. The investigating research materials were approved by the local ethics commission of IGAB, PAS, Jastrzębiec, Poland (permission No. 3/2005). The experimental designs, with detailed laboratory procedures including isolation of total RNA, library preparation and NGS sequencing using the Illumina NextSeq 500 High Output/300 cycle platform and kits (Illumina), were performed as previously described in a recent study [9].

### Bioinformatics analysis

Complete bioinformatics analysis, including read alignment of RNA-seq data to the reference genome, breed-specific SNP detections, QTL/CG analysis, phylogenetic analysis, SNP validation by KASP^TM^ genotyping assay, was performed as previously described in a recent study [9]. However, i) the SNP filtering criterion, ii) the number of QTL/CG loci to investigate the RNA-seq SNP-db, and iii) SNP markers selected for SNP validation by KASP^TM^ genotyping assay were slightly modified from our recent bovine pituitary gland RNA-seq study [9].

#### SNP filtering criterion

To construct the bovine liver SNP-db, stringent parameters of a minimum depth of 10 SNP reads that support the polymorphic nucleotide base with a SNP ratio of 100%, were carried out using Microsoft office excel program in the following manner:

- Stringent parameter of SNP filtering with minimum depth of 10 SNP reads that support the polymorphic nucleotide base,
- Stringent parameter of SNP filtering with a SNP ratio of 100%,
- Combining the stringent parameters of a minimum depth of 10 SNP reads that support the polymorphic nucleotide base with a SNP ratio of 100%.

Using Microsoft office excel program, the bovine liver SNP-dbs of the 18 young bulls were combined according to breed and were further trimmed to one SNP-db that was highly reliable and specific to the three breeds.

### Breed-specific SNP discovery and QTL/CG analysis

Based on a publicly available animal QTL database (http://www.animalgenome.org/cgi-bin/QTLdb/index), a total of 110 potential QTL/CGs (S46 Table) for bovine body growth and developmental trait were included to investigate the RNA-seq SNP-db of bovine liver using Microsoft Office Excel.

#### Phylogenetic analysis

The phylogenetic analysis of the breed-specific SNP-dbs of bovine liver transcriptome was inferred by using the Maximum Likelihood method based on the JTT matrix-based model [28]. The phylogenetic tree(s) of the 18 bull samples representing the Polish Red, Polish HF and Hereford breeds were constructed, firstly with heuristic search by applying Neighbor-Join and BioNJ algorithms to a matrix of pairwise distances estimated using a JTT model, and then by selecting the topology with superior log likelihood value. Finally, the evolutionary analyses were conducted in MEGA7 [29].

### SNP validation by KASP^TM^ genotyping assay

The primer sequences of nine breed-specific SNPs from the Polish Red (n = 2), Polish HF (n = 4), Hereford (n = 3) breeds selected for KASP^TM^ SNP genotyping assay to test the SNP validation experiment are presented in Table 7. The complete methodological procedure of the KASP^TM^ genotyping assay, including statistical analysis using Genepop software (http://genepop.curtin.edu.au) was performed as previously described in a recent study [9].

**Table 7:** SNP mutations and primer sequences of selected SNPs originating from the bovine liver RNA-seq experiment.

SNP-ID	Ref allele	Mutant allele	KASP^TM^ SNP genotyping assay primer sequences
19PR-24970466-CTNS	C	G	TCTCGTAAAGGACCA[C/G]TATTTCCAAACCTT
7PR-23497153-P4HA2	G	A	GTGCTAAAGCAGCTC[G/A]ATGCTGGGGAGGAG
20HF-31891025-GHR	G	A	AGGCTTTCTGTGGTG[G/A]TGTAAATGTCTTCC
9HF-97733752-IGF2R	T	C	AGCTGTCGTCCATCA[C/T]GGGCTCCAGCAGCC
4HF-32078842-IGF2BP3	C	T	TTACTTTGAGGCTCA[C/T]GACAGTGCCTGGCA
19HF-41337061-IGFBP4	G	A	TCATACCCTTGTCTT[G/A]GCAGTGCCACCCGG
20HER-31894358-GHR	A	C	ATGTCTTTGGAGCTA[A/C]TGGAACTCCTCTTT
29HER-50047844-IGF2	C	A	CCCCTCTCCTCTCCC[C/A]CAGGGGACGAAGAG
10HER-7576693-IQGAP2	C	A	ATTTAAAAAAAAAAA[C/A]AAAAAACACATAAA

## Supporting information

S1 Table RNA-seq SNP-db of bovine liver tissue of young bull-1 of Polish Red cattle aged 6 months. (XLSX)

S2 Table RNA-seq SNP-db of bovine liver tissue of young bull-2 of Polish Red cattle aged 6 months. (XLSX)

S3 Table RNA-seq SNP-db of bovine liver tissue of young bull-3 of Polish Red cattle aged 9 months. (XLSX)

S4 Table RNA-seq SNP-db of bovine liver tissue of young bull-4 of Polish Red cattle aged 9 months. (XLSX)

S5 Table RNA-seq SNP-db of bovine liver tissue of young bull-5 of Polish Red cattle aged 12 months. (XLSX)

S6 Table RNA-seq SNP-db of bovine liver tissue of young bull-6 of Polish Red cattle aged 12 months. (XLSX)

S7 Table RNA-seq SNP-db of bovine liver tissue of young bull-7 of Polish HF cattle aged 6 months. (XLSX)

S8 Table RNA-seq SNP-db of bovine liver tissue of young bull-8 of Polish HF cattle aged 6 months. (XLSX)

S9 Table RNA-seq SNP-db of bovine liver tissue of young bull-9 of Polish HF cattle aged 9 months. (XLSX)

S10 Table RNA-seq SNP-db of bovine liver tissue of young bull-10 of Polish HF cattle aged 9 months. (XLSX)

S11 Table RNA-seq SNP-db of bovine liver tissue of young bull-11 of Polish HF cattle aged 12 months. (XLSX)

S12 Table RNA-seq SNP-db of bovine liver tissue of young bull-12 of Polish HF cattle aged 12 months. (XLSX)

S13 Table RNA-seq SNP-db of bovine liver tissue of young bull-13 of Hereford cattle aged 6 months. (XLSX)

S14 Table RNA-seq SNP-db of bovine liver tissue of young bull-14 of Hereford cattle aged 6 months. (XLSX)

S15 Table RNA-seq SNP-db of bovine liver tissue of young bull-15 of Hereford cattle aged 9 months. (XLSX)

S16 Table RNA-seq SNP-db of bovine liver tissue of young bull-16 of Hereford cattle aged 9 months. (XLSX)

S17 Table RNA-seq SNP-db of bovine liver tissue of young bull-17 of Hereford cattle aged 12 months. (XLSX)

S18 Table RNA-seq SNP-db of bovine liver tissue of young bull-18 of Hereford cattle aged 12 months. (XLSX)

S19 Table SNPs filtering data set of Polish Red breed. (XLSX)

S20 Table SNPs filtering data set of Polish HF breed. (XLSX)

S21 Table SNPs filtering data set of Hereford breed. (XLSX)

S22 Table SNPs filtering data set with no errors of Polish Red breed. (XLSX)

S23 Table SNPs filtering data set with no errors of Polish HF breed. (XLSX)

S24 Table SNPs filtering data set with no errors of Hereford breed. (XLSX)

S25 Table SNPs filtering data set with a minimum depth of 10 SNP reads that support the polymorphic nucleotide base of Polish Red breed. (XLSX)

S26 Table SNPs filtering data set with a minimum depth of 10 SNP reads that support the polymorphic nucleotide base of Polish HF breed. (XLSX)

S27 Table SNPs filtering data set with a minimum depth of 10 SNP reads that support the polymorphic nucleotide base of Hereford breed. (XLSX)

S28 Table SNPs filtering data set with SNP ratio = 100% of Polish Red breed. (XLSX)

S29 Table SNPs filtering data set with SNP ratio = 100% of Polish HF breed. (XLSX)

S30 Table SNPs filtering data set with SNP ratio = 100% of Hereford breed. (XLSX)

S31 Table SNPs filtering data set with a minimum depth of 10 SNP reads that support the polymorphic nucleotide base and SNP ratio = 100% of Polish Red breed. (XLSX)

S32 Table SNPs filtering data set with a minimum depth of 10 SNP reads that support the polymorphic nucleotide base and SNP ratio = 100% of Polish HF breed. (XLSX)

S33 Table SNPs filtering data set with a minimum depth of 10 SNP reads that support the polymorphic nucleotide base and SNP ratio = 100% of Hereford breed. (XLSX)

S34 Table Breed comparison in SNPs filtering data set of Polish Red with Polish HF breed. (XLSX)

S35 Table Breed comparison in SNPs filtering data set of Polish Red with Hereford breed. (XLSX)

S36 Table Breed comparison in SNPs filtering data set of Polish Red with Polish HF and Hereford breeds. (XLSX)

S37 Table Breed comparison in SNPs filtering data set of Polish HF with Polish Red breed. (XLSX)

S38 Table Breed comparison in SNPs filtering data set of Polish HF with Hereford breed. (XLSX)

S39 Table Breed comparison in SNPs filtering data set of Polish HF with Polish Red and Hereford breeds. (XLSX)

S40 Table Breed comparison in SNPs filtering data set of Hereford with Polish Red breed. (XLSX)

S41 Table Breed comparison in SNPs filtering data set of Hereford with Polish HF breed. (XLSX)

S42 Table Breed comparison in SNPs filtering data set of Hereford with Polish Red and Polish HF breeds. (XLSX)

S43 Table Identification of De novo SNPs data set in Polish Red breed. (XLSX)

S44 Table Identification of De novo SNPs data set in Polish HF breed. (XLSX)

S45 Table Identification of De novo SNPs data set in Hereford breed. (XLSX)

S46 Table Lists of 110 SNP loci within the CGs bovine growth trait with representing full names, UMD3.1 genome locations, chromosomal locations and web links at bovine QTL-DB. (XLSX)

S47 Table Identification of putative SNPs hits of liver RNA-seq data on 110 potential candidate genes from bovine QTL-db in Polish Red cattle. (XLS)

S48 Table Identification of putative SNPs hits of liver RNA-seq data on 110 potential candidate genes from bovine QTL-db in Polish HF cattle. (XLS)

S49 Table Identification of putative SNPs hits of liver RNA-seq data on 110 potential candidate genes from bovine QTL-db in Hereford cattle. (XLS)

S50 Table Summary of identified putative SNPs hits of liver RNA-seq data on 110 potential candidate genes from bovine QTL-db in Polish Red cattle. (XLS)

S51 Table Summary of identified putative SNPs hits of liver RNA-seq data on 110 potential candidate genes from bovine QTL-db in Polish HF cattle. (XLS)

S52 Table Summary of identified putative SNPs hits of liver RNA-seq data on 110 potential candidate genes from bovine QTL-db in Hereford cattle. (XLS)

S53 Table RNA-seq experimental results of nine selected breed-specific SNPs loci utilized in SNP validation experiment. (XLSX)

S54 Table Primer design details of nine breed-specific SNPs generated from the bovine liver RNA-seq experiment. (XLSX)

S55 Table Distribution of KASP^TM^ genotypes of nine selected breed-specific SNPs originating from the bovine liver RNA-seq experiment. (XLSX)

S56 Table Fixed effect of breeds and developmental ages on validated SNPs markers using REML mixed model procedure. (XLSX)

S57 Table SNPs genotypes and allele frequencies of selected breed-specific SNPs loci in all investigated cattle breeds. (DOC)

S58 Table Genetic differentiation of SNP alleles among investigated cattle breeds using the Fisher's Exact Probability test. (DOC)

S59 Table Genetic differentiation of SNP genotypes among investigated cattle breeds using the Fisher's Exact G test. (DOC)

S60 Table Genetic differentiation comparison of SNP alleles among cattle breeds using the Fisher's Exact Probability test. (DOC)

S61 Table Genetic differentiation comparison of SNP genotypes among cattle breeds using the Fisher's Exact G test. (DOC)

S62 Table Hardy-Weinberg test for genetic differentiation of investigated SNP loci using the Markov chain method. (DOC)

S63 Table Hardy-Weinberg test for genetic differentiation of investigated cattle breeds using the Markov chain method. (DOC)

## Abbreviations

SNP: Single nucleotide polymorphism
RNA-seq: RNA sequencing
HF: Holstein-Friesian
KASP^TM^: kompetitive allele-specific PCR
BWA: Burrows-Wheeler Aligner
MAS: marker assisted selection
GAS: gene assisted selection
GS: genomics selection
QTL: Quantitative trait locus
QTNs: quantitative trait nucleotides
DEGs: differentially expressed genes
CEGs: co-expressed genes
GBS: Genotyping-by-Sequencing
BTA: Bos Taurus autosome
*indel*: insertion and deletion
SNP-db: SNP database
HWE: Hardy Weinberg equilibrium
HT: high-throughput
NGS: next generation sequencing
RIN: RNA Integrity Number
QC: quality control
WGS: whole genome sequence
GWAS: genome-wide association
eQTL: expression quantitative trait loci
TAGs: trait associated genes
*CTNS*: *cystinosin*, *lysosomal cystine transporter*
*P4HA2*: *prolyl 4-hydroxylase subunit alpha 2*
*IQGAP*: *IQ motif containing GTPase* *activating protein homologue*
CGs: candidate genes
*IGF2BP2*: *insulin**-like growth factor 2 binding protein 2*
*GAD1*: *Glutamate* *decarboxylase 1*
*IGFBP2*: *insulin**-like growth factor binding protein 2*
*IGFBP5*: *insulin-like* *growth factor binding protein 5*
*MSTN*: *my**ostatin*
*LEPR*: *leptin* *receptor*
*APOA2*: *apolipoprotein* *A2*
*SLC44A5*: *solute carrier family 44*, *member 5*
*NTNG1*: *netrin* *G1*
*GHRHR*: *growth hormone* *releasing hormone receptor*
*IGF2BP3*: *insulin-like* *growth factor 2 binding protein 3*
*IGFBP1*: *insulin-like* *growth factor binding protein 1*
*IgFBp3*: *insulin-like* *growth factor binding protein 3*
*LEP*: *l**eptin*
*NPY*: *neuropeptide* *Y*
*NSIG1*: *insulin* *Induced gene 1*
*DPP6*: *dipeptidyl* *aminopeptidase-like protein 6*
*HGF*: *hepatocyte* *growth factor*
*IGFBP6*: *insulin-like growth factor binding protein 6*
*IGF-I*: *insulin-like growth factor 1*
*WNT10B*: *wingless-type MMTV integration site family member 10B*
*MYF6*: *myogenic factor 6*
*MYF5*: *myogenic factor 5*
*IGFBP7*: *insulin-like growth factor binding protein 7*
*CCSER1*: *coiled-coil serine-rich protein 1*
*PKD2*: *polycystic kidney disease 2*
*NCAPG*: *non-SMC condensin I complex*, *subunit G*
*KCNIP4*: *kv channel interacting protein 4*
*CAST*: *calpastatin*
*NPM1*: *nucleophosmin*
*PROP1*: *PROP paired-like homeobox 1*
*IGFBPL1*: *insulin-like growth factor binding protein-like 1*
*ATP6V1B2*: *ATPase*, *H+ transporting*, *lysosomal 9kDa*, *V1 subunit B2*
*IGF2R*: *insulin-like growth factor 2 receptor*
*RFX6*: *regulatory factor X*, *6*
*MAP3K5*: *mitogen-activated protein kinase 5*
GTF3C5: *general transcription factor IIIC*, *polypeptide 5*
*RALGDS*: *ras association*
*SDC1*: *syndecan 1*
*DNMT3B*: *DNA*, *(Cytosine-5-)-methyltransferase 3 beta*
*GHRH*: *growth hormone releasing hormone*
*FOXA2*: *forkhead box A2*
*HNF4A*: *hepatocyte nuclear factor 4*, *alpha*
*PLAG1*: *pleiomorphic adenoma gene 1*
*NUCB2*: *nucleobindin 2*
*BTG4*: *B-cell translocation gene 4*
*MGC134087*: *Bos taurus UPF0686 protein C11orf1 homolog-like*, *(LOC538766)*
*ATM*: *ATM Serine/Threonine Kinase*
*UCP3*: *uncoupling protein 3*
*IGFN1*: *immunoglobulin-like and fibronectin type III domain containing 1*
*CAPN2*: *calpain 2*
*PRDM16*: *PR domain containing 16*
*ZBED6*: *zinc finger*, *BED-type containing 6*
*LHX4*: *LIM homeobox 4*
*IGFLR1*: *IGF-like family member 1-like receptor*
*GFL1*: *IGF-like family member 1-like*
*IGFBP4*: *insulin-like growth factor binding protein 4*
*IGF2BP1*: *insulin-like growth factor 2 binding protein 1*
*FASN*: *fatty acid synthase*
*SREBF1*: *sterol regulatory element binding transcription factor 1*
*CARTPT*: *CART prepropeptide*
*GHR*: *growth hormone receptor*
*PRLR*: *prolactin receptor*
*IGF1R*: *insulin-like growth factor 1 receptor*
*GHRL*: *ghrelin/obestatin prepropeptide*
*MST1R*: *macrophage stimulating 1 receptor*
*DLK2*: *delta-like 2 homolog*
*MC4R*: *melanocortin 4 receptor*
*NPC1*: *niemann-pick disease*, *type C1*
*XYLT1*: *xylosyltransferase I*
*SH2B2*: *H2B adaptor protein 2*
*SIRT1*: *sirtuin 1*
*IGF2*: *insulin-like growth factor 2*
*CAPN1*: *calpain 1*
*VEGFB*: *vascular endothelial growth factor B*
*POU1F1*: *POU domain*, *class 1*, *transcription factor 1*
*GHSR*: *Growth hormone secretagogue receptor*
*FABP3*: *Fatty acid binding protein 3*
*NKAIN1*: *Na+/K+ transporting ATPase interacting 1*
*AMPD1*: *Adenosine monophosphate deaminase 1*
*BMP8B*: *Bone morphogenetic protein 8b*
*GRB10*: *Growth factor receptor-bound protein 10*
*HGF*: *Hepatocyte growth factor*, *(hepapoietin A; scatter factor)*
*PEG10*: *paternally expressed gene 10*
*SLC38A1*: *Solute carrier family 38*, *member 1*
*STAT6*: *Signal transducer and activator of transcription 6*
*SPP1*: *Secreted phosphoprotein 1*
*GDF 9*: *Growth differentiation factor 9*
*PCSK1*: *Proprotein convertase subtilisin/kexin type 1*
*CAPN3*: *Calpain 3*
*SIX3*: *SIX homebox 3*
*GDF5*: *Growth differentiation factor 5*
*TG*: *Thyroglobulin*
*XKR4*: *Kell blood group complex subunit-related family*, *member 4*
*SDR16C5*: *Short chain dehydrogenase/reductase family 16C*, *member 5epidermal retinol dehydrogenase 2*
*SDR16C6*: *Short chain dehydrogenase/reductase family 16C*, *member 6 epidermal retinol dehydrogenase 2*
*FAM110B*: *Family with sequence similarity 110*, *member B protein*
*TOX*: *Thymocyte selection-associated high mobility group box*
*CA8*: *Carbonic anhydrase VIII*
*CHD7*: *Chromodomain helicase DNA binding protein 7*
*DGAT1*: *Diacylglycerol O-acyltransferase 1*
*FGF2*: *Fibroblast growth factor 2*
*GH1*: *Somatotrophin*
*NSMCE2*: *non-structural maintenance of chromosomes element 2 homolog*
*MTSS1*: *Metastasis suppressor 1metastasis suppressor protein 1*
*SNTG1*: *Syntrophin*, *gamma 1*
*PPP2R1A*: *Protein phosphatase 2*, *regulatory subunit A*
*EGFR*: *Epidermal growth factor receptor*
*IGFALS*: *Insulin-like growth factor binding protein*, *acid labile subunit*
*GDF10*: *Growth differentiation factor 10*
