# Supplementary material for: Single nucleotide polymorphism discovery in bovine liver using RNA-seq technology
Source: PLoS One. 2017 Feb 24;12(2):e0172687. doi: 10.1371/journal.pone.0172687 (PMC5325534; doi:10.1371/journal.pone.0172687)
Supplement: S57 Table — (DOC) [file pone.0172687.s057.doc]

S57 Table: SNPs genotypes and allele frequencies of selected breed-specific SNPs loci in all investigated cattle breeds.

| Selected SNP marker ID | Distribution of SNP genotypes (n) | | | Allele frequencies (%) | | χ^2^ values |
| --- | --- | --- | --- | --- | --- | --- |
|  | Ref allele | Heterozygous | Mutant allele | Ref Allele | Mutant Allele |  |
| 19PR-24970466-CTNS | 5 | 12 | 27 | 25 | 75 | **3.27*** |
| 7PR-23497153-P4HA2 | 2 | 10 | 32 | 15.91 | 84.09 | 1 |
| 20HF-31891025-GHR | 41 | 3 | 0 | 96.59 | 3.41 | 0.05 |
| 9HF-97733752-IGF2R | 34 | 8 | 2 | 86.36 | 13.64 | **4.58*** |
| 4HF-32078842-IGF2BP3 | 11 | 20 | 13 | 47.73 | 52.27 | 0.35 |
| 20HER-31894358-GHR | 20 | 13 | 11 | 60.23 | 39.77 | **6.46*** |
| 10HER-7576693-IQGAP2 | 4 | 10 | 30 | 20.69 | 79.31 | **7.8*** |
