# Supplementary material for: Single nucleotide polymorphism discovery in bovine liver using RNA-seq technology
Source: PLoS One. 2017 Feb 24;12(2):e0172687. doi: 10.1371/journal.pone.0172687 (PMC5325534; doi:10.1371/journal.pone.0172687)
Supplement: S58 Table — (DOC) [file pone.0172687.s058.doc]

S58 Table: Genetic differentiation of SNP alleles among investigated cattle breeds using the Fisher's Exact Probability test.

| Locus | Breeds | Allele1 | Allele2 | Total | P Value | S.E. |
| --- | --- | --- | --- | --- | --- | --- |
| 19PR-24970466-CTNS | Hereford | 28 | 2 | 30 | 0.00026 | 0.000102119 |
|  | Polish Red | 15 | 15 | 30 |  |  |
|  | Polish HF | 23 | 5 | 28 |  |  |
|  | Total | 66 | 22 | 88 |  |  |
| 7PR-23497153-P4HA2 | Hereford | 1 | 29 | 30 | 0 | 0 |
|  | Polish Red | 12 | 18 | 30 |  |  |
|  | Polish HF | 1 | 27 | 28 |  |  |
|  | Total | 14 | 74 | 88 |  |  |
| 9HF-97733752-IGF2R | Hereford | 22 | 8 | 30 | 0.04794 | 0.00187761 |
|  | Polish Red | 28 | 2 | 30 |  |  |
|  | Polish HF | 26 | 2 | 28 |  |  |
|  | Total | 76 | 12 | 88 |  |  |
| 20HF-31891025-GHR | Hereford | 27 | 3 | 30 | 0.10531 | 0.00181073 |
|  | Polish Red | 30 | 0 | 30 |  |  |
|  | Polish HF | 28 | 0 | 28 |  |  |
|  | Total | 85 | 0 | 88 |  |  |
| 4HF-32078842-IGF2BP3 | Hereford | 23 | 7 | 30 | 0.00363 | 0.000589925 |
|  | Polish Red | 11 | 19 | 30 |  |  |
|  | Polish HF | 12 | 16 | 28 |  |  |
|  | Total | 46 | 42 | 88 |  |  |
| 20HER-31894358-GHR | Hereford | 11 | 19 | 30 | 0.75905 | 0.00391052 |
|  | Polish Red | 11 | 19 | 30 |  |  |
|  | Polish HF | 13 | 15 | 28 |  |  |
|  | Total | 35 | 53 | 88 |  |  |
| 10HER-7576693-IQGAP2 | Hereford | 14 | 16 | 30 | 0.00013 | 8.2456e-05 |
|  | Polish Red | 3 | 25 | 28 |  |  |
|  | Polish HF | 1 | 27 | 28 |  |  |
|  | Total | 18 | 68 | 86 |  |  |
