# Supplementary material for: Single nucleotide polymorphism discovery in bovine liver using RNA-seq technology
Source: PLoS One. 2017 Feb 24;12(2):e0172687. doi: 10.1371/journal.pone.0172687 (PMC5325534; doi:10.1371/journal.pone.0172687)
Supplement: S59 Table — (DOC) [file pone.0172687.s059.doc]

S59 Table: Genetic differentiation of SNP genotypes among investigated cattle breeds using the Fisher's Exact G test.

| Locus | Breeds | Ref | Hetero. | Mutant | Total | P Value | S.E. |
| --- | --- | --- | --- | --- | --- | --- | --- |
| 19PR-24970466-CTNS | Hereford | 13 | 2 | 0 | 15 | 0.00199 | 0.000592971 |
|  | Polish Red | 4 | 7 | 4 | 15 |  |  |
|  | Polish HF | 10 | 3 | 1 | 14 |  |  |
|  | Total | 27 | 12 | 5 | 44 |  |  |
| 7PR-23497153-P4HA2 | Hereford | 0 | 1 | 14 | 15 | 0.00017 | 9.54045e-05 |
|  | Polish Red | 2 | 8 | 5 | 15 |  |  |
|  | Polish HF | 0 | 1 | 13 | 14 |  |  |
|  | Total | 2 | 10 | 32 | 44 |  |  |
| 9HF-97733752-IGF2R | Hereford | 9 | 4 | 2 | 15 | 0.09973 | 0.0030718 |
|  | Polish Red | 13 | 2 | 0 | 15 |  |  |
|  | Polish HF | 12 | 2 | 0 | 14 |  |  |
|  | Total | 34 | 8 | 2 | 44 |  |  |
| 20HF-31891025-GHR | Hereford | 0 | 12 | 3 | 15 | 0.09608 | 0.00178635 |
|  | Polish Red | 0 | 15 | 0 | 15 |  |  |
|  | Polish HF | 0 | 14 | 0 | 14 |  |  |
|  | Total | 0 | 41 |  | 44 |  |  |
| 4HF-32078842-IGF2BP3 | Hereford | 8 | 7 | 0 | 15 | 0.00591 | 0.000648245 |
|  | Polish Red | 1 | 9 | 5 | 15 |  |  |
|  | Polish HF | 4 | 4 | 6 | 14 |  |  |
|  | Total | 13 | 20 | 11 | 44 |  |  |
| 20HER-31894358-GHR | Hereford | 4 | 3 | 8 | 15 | 0.82407 | 0.00260339 |
|  | Polish Red | 4 | 3 | 8 | 15 |  |  |
|  | Polish HF | 3 | 7 | 4 | 14 |  |  |
|  | Total | 11 | 13 | 20 | 44 |  |  |
| 10HER-7576693-IQGAP2 | Hereford | 4 | 6 | 5 | 15 | 0.00044 | 0.000125786 |
|  | Polish Red | 0 | 3 | 11 | 14 |  |  |
|  | Polish HF | 0 | 1 | 13 | 14 |  |  |
|  | Total | 4 | 10 | 29 | 43 |  |  |
