# Supplementary material for: Single nucleotide polymorphism discovery in bovine liver using RNA-seq technology
Source: PLoS One. 2017 Feb 24;12(2):e0172687. doi: 10.1371/journal.pone.0172687 (PMC5325534; doi:10.1371/journal.pone.0172687)
Supplement: S60 Table — (DOC) [file pone.0172687.s060.doc]

S60 Table: Genetic differentiation comparison of SNP alleles among cattle breeds using the Fisher's Exact Probability test.

| Locus | | Breed comparison | | P Value | S.E. |
| --- | --- | --- | --- | --- | --- |
| 19PR-24970466-CTNS | | Polish Red vs Hereford | | 0.00020 | 0.00009 |
|  |  | Polish HF vs Hereford | | 0.25072 | 0.00274 |
|  |  | Polish HF vs Polish Red | | 0.01364 | 0.00080 |
| 7PR-23497153-P4HA2 | | Polish Red vs Hereford | | 0.00103 | 0.00020 |
|  |  | Polish HF vs Hereford | | 1.00000 | 0.00000 |
|  |  | Polish HF vs Polish Red | | 0.00143 | 0.00023 |
| 9HF-97733752-IGF2R | | Polish Red vs Hereford | | 0.07579 | 0.00172 |
|  |  | Polish HF vs Hereford | | 0.08074 | 0.00169 |
|  |  | Polish HF vs Polish Red | | 1.00000 | 0.00000 |
| 20HF-31891025-GHR | | Polish Red vs Hereford | | 0.23948 | 0.00189 |
|  |  | Polish HF vs Hereford | | 0.23895 | 0.00202 |
|  |  | Polish HF vs Polish Red | | - | - |
| 4HF-32078842-IGF2BP3 | | Polish Red vs Hereford | | 0.00362 | 0.00054 |
|  |  | Polish HF vs Hereford | | 0.01422 | 0.00116 |
|  |  | Polish HF vs Polish Red | | 0.78977 | 0.00189 |
| 20HER-31894358-GHR | | Polish Red vs Hereford | | 1.00000 | 0.00000 |
|  |  | Polish HF vs Hereford | | 0.59731 | 0.00316 |
|  |  | Polish HF vs Polish Red | | 0.58832 | 0.00332 |
| 10HER-7576693-IQGAP2 | | Polish Red vs Hereford | | 0.00376 | 0.00042 |
|  |  | Polish HF vs Hereford | | 0.00027 | 0.00012 |
|  |  | Polish HF vs Polish Red | | 0.61136 | 0.00181 |
| Overall significance of SNP alleles for each population pair (cattle breeds) across all loci using Fisher's exact Probability test | | | | | |
| Locus | breed comparison | |  χ2 value | df | P Value |
| All loci | Polish Red vs Hereford | | 61.21816 | 14 | 0.000000 |
| All loci | Polish HF vs Hereford | | 36.633908 | 14 | 0.000838 |
| All loci | Polish HF vs Polish Red | | 24.206793 | 14 | 0.019063 |
