# Supplementary material for: Single nucleotide polymorphism discovery in bovine liver using RNA-seq technology
Source: PLoS One. 2017 Feb 24;12(2):e0172687. doi: 10.1371/journal.pone.0172687 (PMC5325534; doi:10.1371/journal.pone.0172687)
Supplement: S61 Table — (DOC) [file pone.0172687.s061.doc]

S61 Table: Genetic differentiation comparison of SNP genotypes among cattle breeds using the Fisher's Exact G test.

| Locus | | Breed comparison | | | P Value | S.E. |
| --- | --- | --- | --- | --- | --- | --- |
| 19PR-24970466-CTNS | | Polish Red vs Hereford | | | 0.00142 | 0.00022 |
|  |  | Polish HF vs Hereford | | | 0.29848 | 0.00293 |
|  |  | Polish HF vs Polish Red | | | 0.02707 | 0.00134 |
| 7PR-23497153-P4HA2 | | Polish Red vs Hereford | | | 0.00123 | 0.00029 |
|  |  | Polish HF vs Hereford | | | 1.00000 | 0.00000 |
|  |  | Polish HF vs Polish Red | | | 0.00088 | 0.00023 |
| 9HF-97733752-IGF2R | | Polish Red vs Hereford | | | 0.13128 | 0.00195 |
|  |  | Polish HF vs Hereford | | | 0.13186 | 0.00234 |
|  |  | Polish HF vs Polish Red | | | 1.00000 | 0.00000 |
| 20HF-31891025-GHR | | Polish Red vs Hereford | | | 0.22252 | 0.00184 |
|  |  | Polish HF vs Hereford | | | 0.22826 | 0.00193 |
|  |  | Polish HF vs Polish Red | | | 0.00000 | 0.00000 |
| 4HF-32078842-IGF2BP3 | | Polish Red vs Hereford | | | 0.00152 | 0.00022 |
|  |  | Polish HF vs Hereford | | | 0.02616 | 0.00125 |
|  |  | Polish HF vs Polish Red | | | 0.79761 | 0.00185 |
| 20HER-31894358-GHR | | Polish Red vs Hereford | | | 1.00000 | 0.00000 |
|  |  | Polish HF vs Hereford | | | 0.64730 | 0.00287 |
|  |  | Polish HF vs Polish Red | | | 0.64761 | 0.00256 |
| 10HER-7576693-IQGAP2 | | Polish Red vs Hereford | | | 0.00865 | 0.00064 |
|  |  | Polish HF vs Hereford | | | 0.00125 | 0.00033 |
|  |  | Polish HF vs Polish Red | | | 0.59622 | 0.00148 |
| Overall significance of SNP genotypes for each population pair (cattle breeds) across all loci using Fisher's exact G test | | | | | | |
| Locus | breed comparison | |  χ2 value | df | P Value | |
| All loci | Polish Red vs Hereford | | 56.06048 | 14 | 0.000001 | |
| All loci | Polish HF vs Hereford | | 30.950835 | 14 | 0.005632 | |
| All loci | Polish HF vs Polish Red | | 23.645331 | 12 | 0.022721 | |
