# Supplementary material for: Single nucleotide polymorphism discovery in bovine liver using RNA-seq technology
Source: PLoS One. 2017 Feb 24;12(2):e0172687. doi: 10.1371/journal.pone.0172687 (PMC5325534; doi:10.1371/journal.pone.0172687)
Supplement: S62 Table — (DOC) [file pone.0172687.s062.doc]

S62 Table: Hardy-Weinberg test for genetic differentiation of investigated SNP loci using the Markov chain method.

| Locus | Breeds | P values | S.E. | df | Chi2 | Prob. |
| --- | --- | --- | --- | --- | --- | --- |
| 19PR-24970466-CTNS | Hereford | 1.0000 | 0.0000 | 6 | 2.1130 | 0.9090 |
|  | Polish Red | 1.0000 | 0.0000 |  |  |  |
|  | Polish HF | 0.3477 | 0.0023 |  |  |  |
| 7PR-23497153-P4HA2 | Hereford | 0.0000 | 0.0000 | - | 0.00000 | 0.00000 |
|  | Polish Red | 1.0000 | 0.0000 |  |  |  |
|  | Polish HF | 0.0000 | 0.0000 |  |  |  |
| 9HF-97733752-IGF2R | Hereford | 0.2229 | 0.0023 | 6 | 3.0023 | 0.8086 |
|  | Polish Red | 1.0000 | 0.0000 |  |  |  |
|  | Polish HF | 1.0000 | 0.0000 |  |  |  |
| 20HF-31891025-GHR | Hereford | 1.0000 | 0.0000 | - | 0.00000 | 0.00000 |
|  | Polish Red | 0.0000 | 0.0000 |  |  |  |
|  | Polish HF | 0.0000 | 0.0000 |  |  |  |
| 4HF-32078842-IGF2BP3 | Hereford | 0.5273 | 0.0014 | 6 | 6.4418 | 0.3756 |
|  | Polish Red | 0.5764 | 0.0019 |  |  |  |
|  | Polish HF | 0.1313 | 0.0019 |  |  |  |
| 20HER-31894358-GHR | Hereford | 0.0349 | 0.0011 | 6 | 13.4874 | 0.0359 |
|  | Polish Red | 0.0338 | 0.0011 |  |  |  |
|  | Polish HF | 1.0000 | 0.0000 |  |  |  |
| 10HER-7576693-IQGAP2 | Hereford | 0.6056 | 0.0018 | 4 | 1.0032 | 0.9093 |
|  | Polish Red | 1.0000 | 0.0000 |  |  |  |
|  | Polish HF | - | - |  |  |  |
