# Supplementary material for: Single nucleotide polymorphism discovery in bovine liver using RNA-seq technology
Source: PLoS One. 2017 Feb 24;12(2):e0172687. doi: 10.1371/journal.pone.0172687 (PMC5325534; doi:10.1371/journal.pone.0172687)
Supplement: S63 Table — (DOC) [file pone.0172687.s063.doc]

S63 Table: Hardy-Weinberg test for genetic differentiation of investigated cattle breeds using the Markov chain method.

| Breeds | Locus | P values | S.E. | | df | χ2 | Probability |
| --- | --- | --- | --- | --- | --- | --- | --- |
| Hereford | 19PR-24970466-CTNS | 1.0000 | 0.0000 | | 12 | 11.9977 | 0.4459 |
|  | 7PR-23497153-P4HA2 | 0.0000 | 0.0000 | |  |  |  |
|  | 9HF-97733752-IGF2R | 0.2229 | 0.0023 | |  |  |  |
|  | 20HF-31891025-GHR | 1.0000 | 0.0000 | |  |  |  |
|  | 4HF-32078842-IGF2BP3 | 0.5273 | 0.0014 | |  |  |  |
|  | 20HER-31894358-GHR | 0.0349 | 0.0011 | |  |  |  |
|  | 10HER-7576693-IQGAP2 | 0.0656 | 0.0018 | |  |  |  |
| Polish Red | 19PR-24970466-CTNS | 1.0000 | 0.0000 | | 12 | 7.8769 | 0.7947 |
|  | 7PR-23497153-P4HA2 | 1.0000 | | 0.0000 |  |  |  |
|  | 9HF-97733752-IGF2R | 1.0000 | | 0.0000 |  |  |  |
|  | 20HF-31891025-GHR | 0.0000 | | 0.0000 |  |  |  |
|  | 4HF-32078842-IGF2BP3 | 0.5764 | | 0.0019 |  |  |  |
|  | 20HER-31894358-GHR | 0.0338 | | 0.0011 |  |  |  |
|  | 10HER-7576693-IQGAP2 | 1.0000 | | 0.0000 |  |  |  |
| Polish HF | 19PR-24970466-CTNS | 0.3477 | 0.0023 | | 8 | 6.1731 | 0.6279 |
|  | 7PR-23497153-P4HA2 | 0.0000 | 0.0000 | |  |  |  |
|  | 9HF-97733752-IGF2R | 1.0000 | 0.0000 | |  |  |  |
|  | 20HF-31891025-GHR | 0.0000 | 0.0000 | |  |  |  |
|  | 4HF-32078842-IGF2BP3 | 0.1313 | 0.0019 | |  |  |  |
|  | 20HER-31894358-GHR | 1.0000 | 0.0000 | |  |  |  |
|  | 10HER-7576693-IQGAP2 | 0.0000 | 0.0000 | |  |  |  |
